# Effect of a one time guideline based educational video intervention on osteoporosis related knowledge

**DOI:** 10.1007/s00402-026-06293-5

**Published:** 2026-04-17

**Authors:** Anna-Lena Hauser, Javier-Fernando Noriega Urena, Levan Saatashvili, Firas Esmail, Tobias Lange, Tobias Ludger Schulte, Alexander von Glinski

**Affiliations:** Orthopedic University Clinic at St. Josef Hospital Bochum, University Hospitals of the Ruhr-University of Bochum, Bochum, Germany

**Keywords:** Osteoporosis, Patient education, Health literacy, Video-based intervention, Osteoporosis knowledge, Randomized controlled trial

## Abstract

**Introduction:**

Osteoporosis remains underdiagnosed and undertreated, partly due to insufficient patient knowledge. This study aimed to assess osteoporosis-related knowledge and to evaluate the immediate and short-term effects of a one-time educational intervention through a guideline-based video in an adult online population.

**Materials and methods:**

In this prospective, randomized controlled trial, adults aged ≥ 18 years were recruited via an established online panel. Participants were randomized to an intervention group, which viewed a standardized educational video on osteoporosis, or a control group without intervention. Osteoporosis knowledge was assessed using the Facts on Osteoporosis Quiz (FOOQ) and the Osteoporosis Knowledge Assessment Tool (OKAT). Knowledge retention was evaluated in the intervention group after one week. Between-group comparisons were performed using Man-Whitney U tests, χ² tests, and odds ratios.

**Results:**

A total of 513 participants were included (intervention group: *n* = 198; control group: *n* = 315), with no significant differences in baseline demographic or clinical characteristics. Immediately after the intervention, osteoporosis-related knowledge was significantly higher in the intervention group compared with controls, as measured by both OKAT (11.6 vs. 8.8 points, *p* < 0.0001) and FOOQ (13.2 vs. 11.1 points, *p* < 0.0001). The proportion of participants with poor knowledge (FOOQ ≤ 10 points) was lower in the intervention group (20.7% vs. 41.3%; OR 0.37, 95% CI 0.24–0.56), while good knowledge (FOOQ ≥ 15 points) was more frequent (37.9% vs. 17.5%; OR 2.88, 95% CI 1.92–4.30). At 1-week follow-up (*n* = 65), knowledge scores in the intervention group remained significantly higher than baseline control values for both FOOQ and OKAT (all *p* < 0.005), indicating short-term knowledge retention.

**Conclusion:**

A brief, guideline-based educational video significantly improves osteoporosis-related knowledge, with substantial retention after one week. Video-based education represents an effective, scalable tool to enhance patient understanding of osteoporosis and may support future prevention and management strategies.

## Introduction

Osteoporosis, a debilitating disease causing reduced bone mass and disruption of bone microarchitecture [1] and resulting in an increased susceptibility to fractures [2], affects nearly 28 million people in Europe [1]. Commonly called a “silent disease” since bone loss progresses asymptomatically until fractures occur, the disease oftentimes progresses to extended damage before diagnosis [3]. Its effects are most visible in aging populations [4] and, as life expectancy rises, its prevalence is constantly increasing [5]. About 9 million fractures annually worldwide are caused by osteoporosis [1]. Osteoporotic fractures cause significant morbidity with patients experiencing prolonged recovery periods, permanent functional limitations [6] and excess mortality extending up to 10 years post fracture [7, 8]. The impact of fractures on patients‘ quality of life and independence is substantial [3, 4].

Osteoporosis therapy encompasses non pharmacological and pharmacological interventions [9]. Both preventive and pharmacological strategies are effective in reducing fracture risk when appropriately implemented [9–15]. Astonishingly, treatment rates not only remain low [9, 16, 17] but are reported to even decline [18]. Reasons might be polypharmacy and drug interactions leading to patient compliance concerns [16] and information gaps regarding the necessity and efficacy of medication [19]. Treatment rates following fracture have been reported to range widely, with many studies showing rates below 10% [20].

This has implications not only for affected individuals but also for healthcare systems as a whole as the cost of osteoporosis is anticipated to rise significantly in the coming decades [21] and, without timely interventions, is projected to become one of the most expensive economic burdens on healthcare systems [22]. The foremost critical intervention is addressing the treatment gap as improved adherence to medication can significantly reduce fracture incidence [23]. Several studies have demonstrated relevant knowledge gaps regarding osteoporosis across different populations. Sitati et al. [24] reported limited awareness among postmenopausal women in Kenya, particularly regarding modifiable risk factors. As postmenopausal women represent a high-risk group for osteoporosis—approximately 80% of all osteoporosis cases affect this population—the study underscores the need for additional educational programs. Edelstein et al. [25] found moderate levels of osteoporosis-related knowledge among male participants in Israel, with higher knowledge being associated with healthier behaviors. Tardi et al. [26] identified age, occupation and educational level as significant determinants of osteoporosis knowledge.

Osteoporosis-related knowledge can be assessed using the Osteoporosis Knowledge Assessment Tool (OKAT), which consists of 20 statements answered as “true,” “false,” or “I don’t know.” Each correct response is awarded one point, yielding a total score ranging from 0 to 20, with higher scores indicating greater osteoporosis-related knowledge. Although the OKAT is not formally normalized, scores between 15 and 20 points have commonly been interpreted as reflecting good knowledge in previous studies [27]. In addition, the Facts on Osteoporosis Quiz (FOOQ) is a widely used instrument comprising 20 items that primarily assess factual knowledge of osteoporosis. Total scores range from 0 to 20, with higher values representing better factual understanding of the disease [28].

Improved patient knowledge has been associated with better adherence to pharmacological therapy [29, 30]. Educational interventions range from face-to-face counselling, brochures, letters or telephone calls [31, 32] to multicomponent interventions consisting of a mixtures of e.g. cognitive-behavioural techniques, motivational interviewing and pharmaceutical care [33–36]. Patients’ values and preferences heavily influence treatment choice and adherence, making decision-making regarding therapeutics a process that encompasses not only the identification of the best treatment strategy but also the incorporation of individual patient factors [37].

In routine clinical practice, which is frequently characterized by limited time, personnel, and resources, such complex educational strategies are difficult to implement at scale. Single educational encounters therefore represent an attractive alternative. However, studies suggest that patients often retain only a limited amount of medical information from single encounters [38] indicating that knowledge retention may be suboptimal without reinforcement. Digital educational tools, including smartphone- and tablet-based applications, offer a scalable and low-threshold approach to patient education and may facilitate both initial knowledge acquisition and short-term retention. Video-based formats, in particular, allow standardized delivery of guideline-concordant information and can be accessed flexibly by patients.

Although increased knowledge alone does not guarantee behavioral change, it represents a prerequisite for informed decision-making and shared decision-making processes. Knowledge should therefore be considered a proximal outcome that may support subsequent preventive and therapeutic strategies.

Given the persistent osteoporosis treatment gap and documented knowledge deficits, there is a need for scalable, low-threshold educational strategies. The present study therefore aimed to assess osteoporosis-related knowledge in an adult online population and to evaluate the immediate and short-term effects of a guideline-based educational video.

## Methods

### Study design

**Fig. 1:**
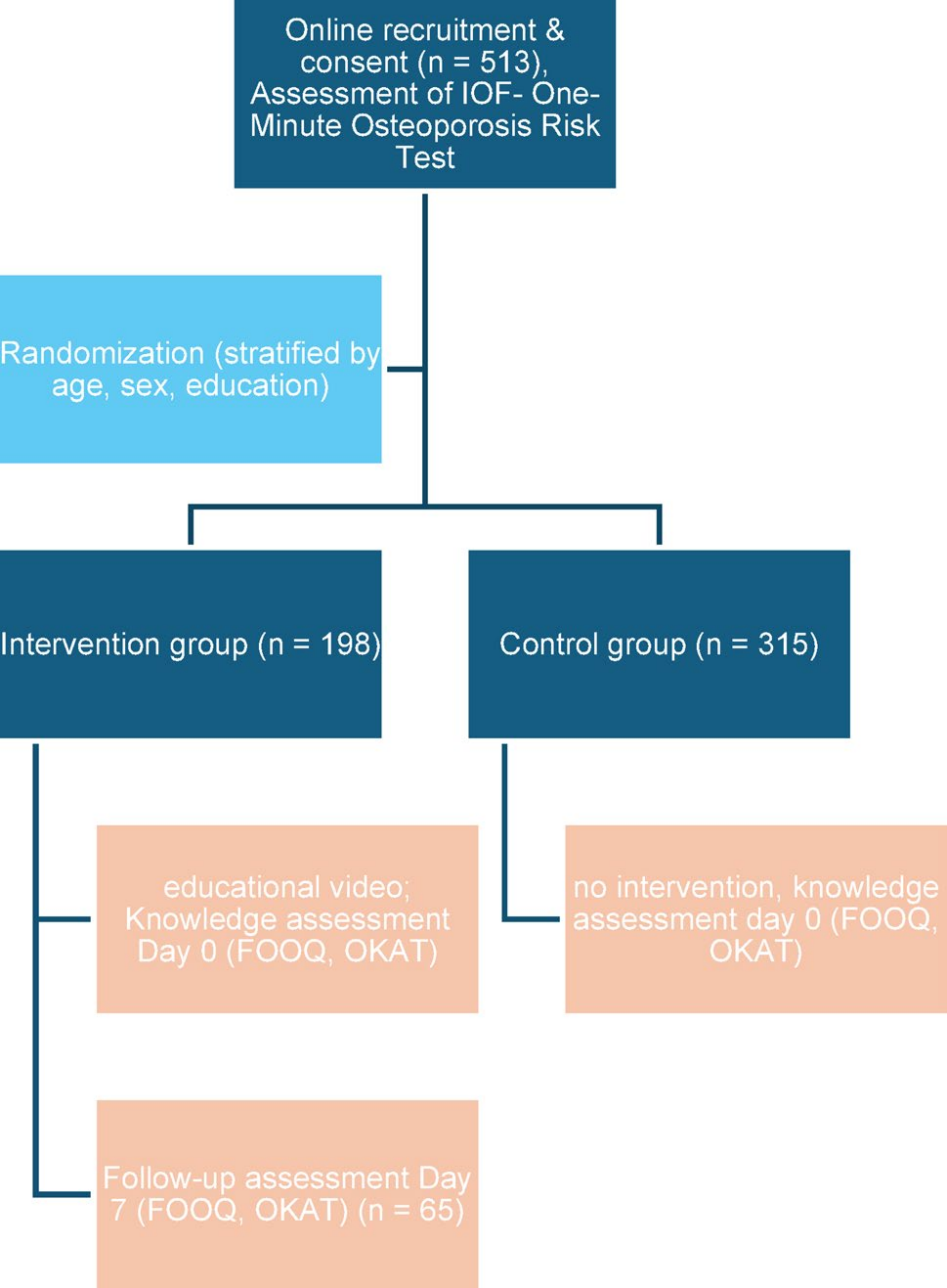
Study design

This study was designed as a prospective, randomized controlled trial conducted within an established online panel (“Mein-Rücken-und-Ich”). The objective was to assess osteoporosis-related knowledge and to evaluate the immediate and short-term effects of a video-based educational intervention. Participants were allocated to either an intervention group or a control group using stratified randomization based on age, sex, and educational level. The control group completed all assessments without exposure to the educational intervention. In the intervention group, knowledge was assessed immediately after the intervention (day 0) and again 7 days later (Fig. 1).

### Participants and recruitment

Eligible participants were adults aged ≥ 18 years who provided electronic informed consent and voluntarily registered via the online platform. Participation was anonymous. Exclusion criteria were incomplete questionnaire responses.

### Intervention

The intervention consisted of a standardized, guideline-based educational video covering osteoporosis pathophysiology, risk factors, prevention, diagnosis, and treatment in lay language. The content was developed by clinical experts and aligned with current German osteoporosis guidelines (AWMF Register No. 183/0019).

### Outcomes

The primary outcome was osteoporosis-related knowledge assessed using two validated instruments: the Facts on Osteoporosis Quiz (FOOQ) and the Osteoporosis Knowledge Assessment Tool (OKAT). The secondary outcome was change in knowledge following the intervention. Osteoporosis risk factors were assessed descriptively using the International Osteoporosis Foundation (IOF) One-Minute Osteoporosis Risk Test.

### Data collection and statistical analysis

All data were collected electronically. Due to the anonymous study design, individual-level linkage between baseline and follow-up responses was not possible. Consequently, all comparisons between day 0 and day 7 represent unpaired group-level analyses. Continuous variables are presented as mean ± standard deviation and categorical variables as number (percentage). Between-group comparisons were performed using Mann-Whitney-U tests or χ² tests, as appropriate. Odds ratios with 95% confidence intervals were calculated for categorical knowledge outcomes. As this was an exploratory study, no formal sample size calculation was performed. Statistical significance was defined as *p* < 0.05.

### Ethics

The trial was approved by the responsible ethics committee (No. 21-7292) and conducted in accordance with the Declaration of Helsinki. All participants provided electronic informed consent prior to participation.

## Results

A total of 513 participants were included in the analysis. Group 1 (intervention group) comprised 198 participants and Group 2 (control group) comprised 315 participants. Demographic and clinical characteristics were comparable between groups. There were no statistically significant differences with respect to age, sex distribution, educational level, employment in a medical profession, prevalence of osteoporosis, or positive family history of osteoporosis (all *p* > 0.05, v. Table 1).

**Table 1:** Demographic and clinical characteristics

	Group 1 *n* = 198	Group 2 *n* = 315
Age	60.54 ± 11.70 years	61.6 ± 12.0 years
Sex distribution	Women: *n* = 160 (80.8%)	Women: *n* = 234 (74.3%)
	Men: *n* = 38 (19.2%)	Men: *n* = 80 (25.4%)
		Non-binary: *n* = 1 (0.3%)
Educational level		
- Hauptschulabschluss	*n* = 13 (6.6%)	- *n* = 18 (5.7%)
- Realschulabschluss	*n* = 52 (26.3%)	- *n* = 93 (29.5%)
- Fachhochschulreife	*n* = 34 (17.2%)	- *n* = 45 (14.3%)
- Allgemeine Hochschulreife	*n* = 100 (50.5%)	- *n* = 158 (50.2%)
- Alternative educational attainment	*n* = 5 (2.5%)	- *n* = 1 (0.3%)
Employment in a medical profession	*n* = 38 (19.2%)	*n* = 52 (16.5%)
prevalence of osteoporosis	*n* = 37 (18.7%)	*n* = 52(16.5%)
positive family history	*n* = 75 (37.9%)	*n* = 119 (37.8%)

Hauptschulabschluss = lower secondary education, Realschulabschluss= intermediate secondary education, Fachhochschulreife = higher education entrance qualification, Allgemeine Hochschulreife = general university entrance qualification

**Fig. 2:**
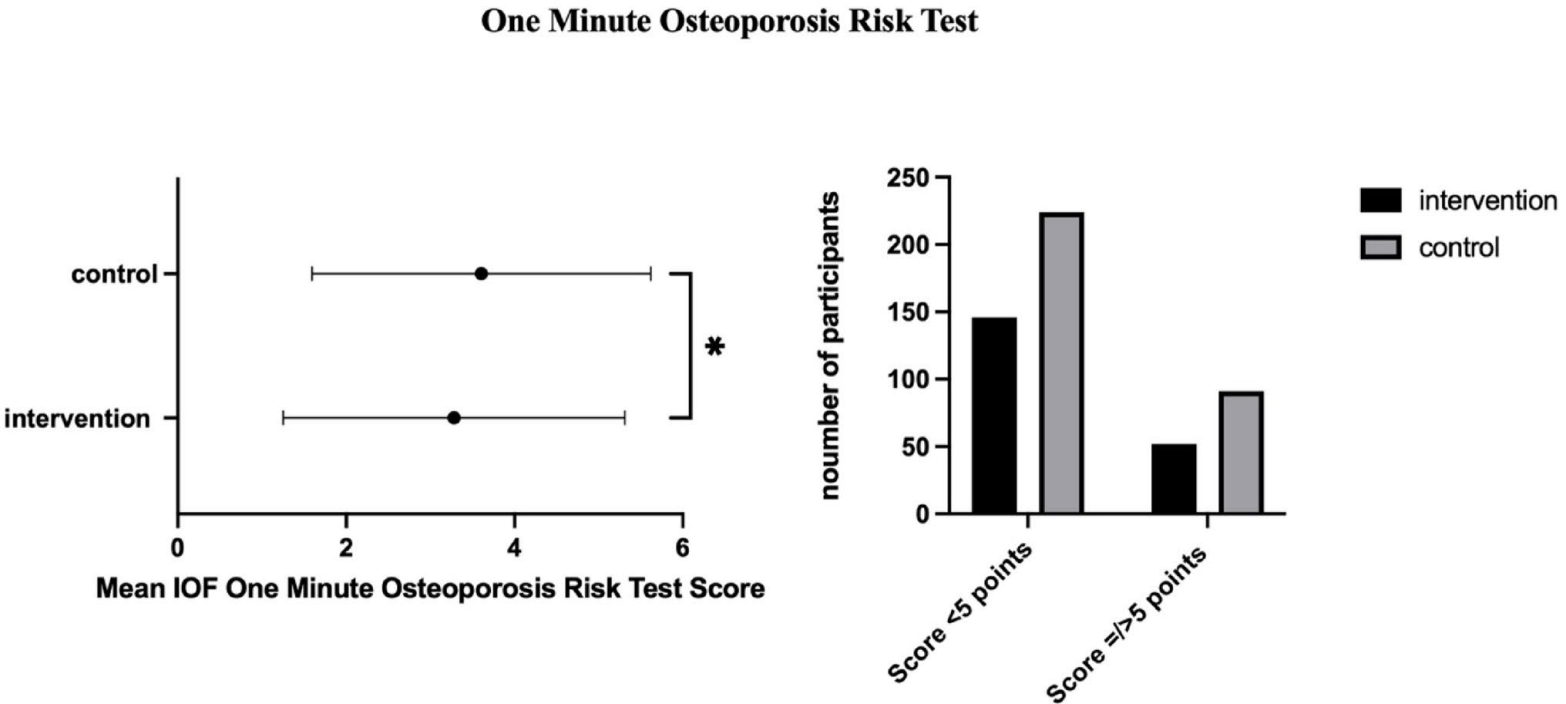
Mean IOF One Minute Osteoporosis Risk Test results; left: mean scores; right: proportion of participants scoring 5 points or higher and thus, per definition, being at risk for osteoporosis

Responses to the IOF One Minute Osteoporosis Risk Test were largely comparable between groups. While there was no statistically significant difference between the mean age of both groups, Group 2 reported a significantly higher prevalence of age ≥ 40 years (*p* < 0.001). This reflects a high overall prevalence of participants aged ≥ 40 years in both groups, resulting in comparable mean ages despite differences in categorical age distribution. Additionally, the frequency of falls/fear of falling (*p* < 0.01) as well as the incidence of current or former smoking was significantly more prevalent than in Group 1. Despite there being no statistically significant between-group differences for the remaining risk factors (all *p* > 0.05), this resulted in significantly different IOF One-Minute Osteoporosis Risk Test scores (Group 1: 3.28±2.03 points, Group 2: 3.61±2.01 points, *p* < 0.05). This difference was primarily driven by small variations across multiple risk items and did not translate into a significantly higher proportion of participants exceeding the predefined high-risk threshold of ≥ 5 points (Group 1: 52 participants (26.3%), Group 2: 91 participants (30.8%); *p* > 0.05, OR = 0.80, 95% CI = 0.53 to 1.20) (Fig 2).

### Test scores on day 0

Directly after watching the video, the Group 1 scored a mean OKAT-Score of 11.56 ± 2.62 points, which was significantly (*p* < 0.0001) higher than in Group 2 with a mean of 8.81 ± 3.06 points.

**Fig. 3:**
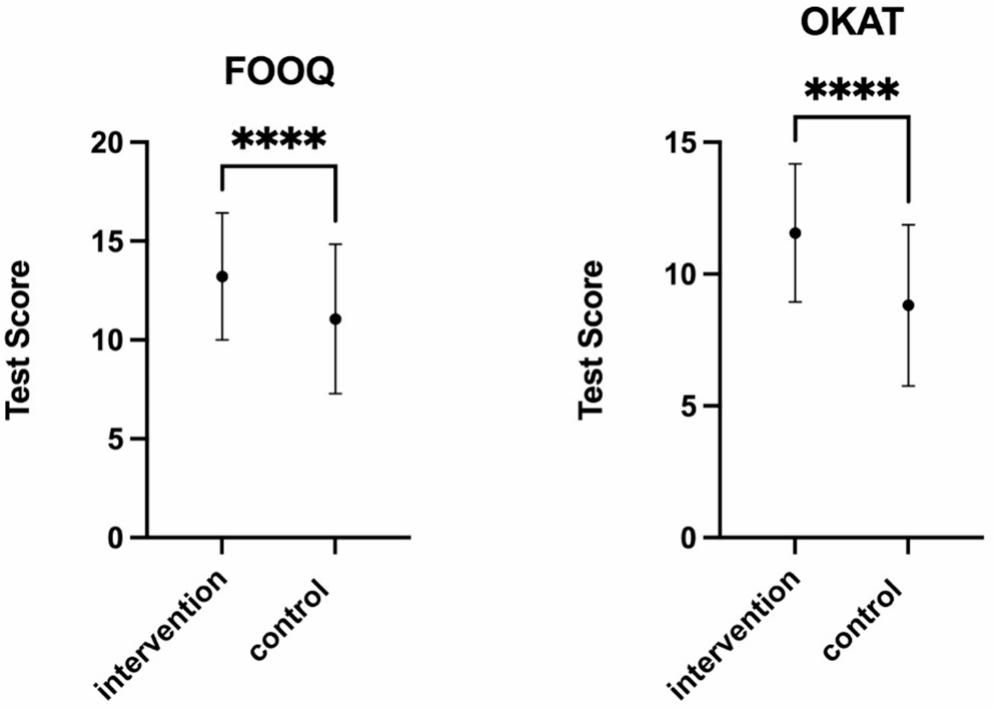
FOOQ and OKAT test scores on day 0. **** = *p* < 0.0001; Group 1 = intervention, Group 2 = control

Group 1 also scored significantly higher on the FOOQ than Group 2 (13.21 ± 3.21 vs. 11.06 ± 3.78 points, *p* < 0.0001). Their risk of scoring 10 points or less and therefore showing poor knowledge was significantly smaller than Group 2’s risk (OR 0.37, 95% CI 0.24 to 0.56, *p* < 0.0001). In absolute terms, 41/198 participants (20.7%) in the Group 1 and 130/315 participants (41.3%) in Group 2 scored ≤ 10 points. Simultaneously their chance of scoring 15 points or higher and therefore showing good knowledge was significantly greater (OR 2.88, 95% CI 1.92 to 4.30, *p* < 0.0001). Correspondingly, 75/198 participants (37.9%) in Group 1 and 55/315 participants (17.5%) in Group 2 scored ≥ 15 points (Fig 3).

### Retest after 1 week

65 patients from the Group 1 (32.8%) completed the 1-week-follow-up questionnaire. Due to the anonymous study design, individual-level linkage between day 0 and day 7 responses was not possible; all follow-up analyses therefore represent unpaired group-level comparisons. No follow-up assessment was performed in the Group 2.

**Fig. 4:**
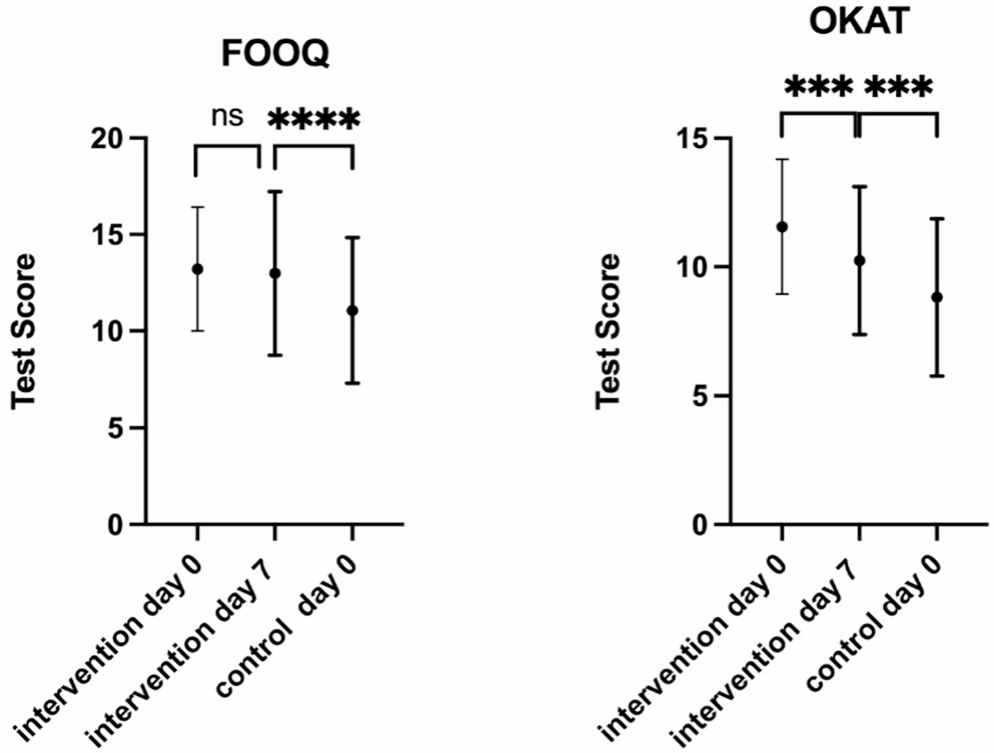
left: FOOQ-Test-Scores; right: OKAT-Test-Scores; **** = *p* < 0.0001; ***= *p* < 0.0005; Group 1 = intervention, Group 2 = control

Group 1 scored a mean of 12.98 ± 4.22 points on the FOOQ on day 7, which was not significantly less than what Group 1 scored on day 0 (*p* > 0.5), but significantly more than Group 2’s result on day 0 (*p* < 0.00001). With a mean OKAT-Score of 10.25 ± 2.88 points they showed a statistically significant decrease compared with day 0 (*p* < 0.005), but still significantly better than Group 2 on day 0(*p* < 0.005) (Fig 4).

## Discussion

In this randomized controlled trial, a single, guideline-based educational video significantly improved patient knowledge regarding osteoporosis. Additionally, substantial knowledge retention was observed one week after exposure, indicating that even low-threshold, time-efficient educational formats may achieve meaningful short-term effects on patient knowledge.

Previous research has repeatedly reported knowledge and education gaps regarding osteoporosis across different patient populations, socioeconomic groups, gender, cultural backgrounds and ages and linked them to poorer therapy adherence. To bridge this gap, recent publications advised to use multicomponent interventions involving repeated interactions between patients and healthcare providers. Especially in public healthcare systems the aggravating problem of an increasing shortage of healthcare providers facing growing numbers of ageing patients and thus a scarcity of time, personnel and resources might explain why complex measures are rarely implemented in routine clinical practice. Our findings suggest that providing informational, guideline-based video information, for example, before physician appointments is associated with increased knowledge and knowledge retention without the use of more resource-intensive educational formats.

In patients with predefined risk factors for osteoporosis, such as prior fractures or glucocorticoid use, the umbrella organization of the German-speaking Scientific Societies for Osteology (DVO) recommends further diagnostic evaluation. This includes bone density assessment using dual-energy X-ray absorptiometry (DXA), baseline laboratory testing, and radiological assessment for prevalent fractures, particularly vertebral fractures [9]. These measures aim to enable early identification of patients at increased fracture risk and to guide subsequent therapeutic decision-making. Mori et al. have suggested that, despite higher radiation exposure, lower trabecular bone mineral density measured by quantitative computed tomography (QCT) is more strongly associated with osteoporotic fracture risk than T-scores obtained by DXA. QCT may therefore offer advantages in selected patient populations, including those with secondary osteoporosis due to glucocorticoid therapy, high fracture risk, or severe obesity, where bone density may be underestimated by DXA [39]. In contrast to established screening programs such as breast cancer screening, patients are not routinely invited but must actively seek guidance from their primary care provider, which requires a certain level of knowledge.

Following diagnostic evaluation, baseline treatment is recommended for all patients. This includes normalization of body mass index (BMI) and adequate protein intake, implementation of regular physical activity and fall prevention strategies, restriction of nicotine and alcohol consumption, and supplementation with vitamin D and calcium [9]. However, evidence suggests that these basic measures are frequently underutilized in clinical practice. Falk et al. reported substantial care gaps, with 84% of patients not receiving basic therapy and 93% not receiving specific pharmacological treatment in a cohort of 102 patients with wrist fractures. Notably, more than half of these patients had already met criteria for treatment prior to the fracture, yet 53% had never undergone screening [40]. In the context of a high prevalence of vitamin D deficiency—even during summer months—this highlights a persistent and clinically relevant gap between guideline recommendations and real-world implementation [40]. Interestingly, patients who self-supplemented vitamin D showed significantly higher serum levels, suggesting a potential association between increased knowledge and health-related behavior [40]. Specific pharmacological treatment is recommended for patients with a 3% risk of vertebral or femoral neck fractures within three years, particularly in the presence of very high fracture risk or irreversible risk factors. Treatment should be initiated in patients with a fracture risk of 5% or greater. Osteoanabolic agents may be considered from a 5% risk threshold and are recommended for patients with a risk of 10% or higher [9].

One might argue that an increase in knowledge alone does not suffice for behavioral change as education alone does not address the patient’s contextual and social influences or monetary resources [41]. It nonetheless represents a necessary, albeit arguably not sufficient, prerequisite for informed decision making and shared decision making processes. Patients who routinely do not experience symptoms before fractures occur and additionally lack a basic understanding of osteoporosis, its risk factors, and available treatment options are unlikely to engage meaningfully in preventive strategies or long-term therapy. Knowledge should not be considered an endpoint in osteoporosis education, but as a proximal outcome that may support subsequent clinical decisions, although this was not directly assessed in the present study, and therefore be considered an important prerequisite for building therapeutic alliances.

Interestingly, knowledge retention differed between FOOQ and OKAT, with FOOQ scores remaining largely stable at one-week follow-up, while OKAT scores showed a modest but statistically significant decrease compared with immediate post-intervention values. This discrepancy may reflect differences in item structure, with OKAT placing greater emphasis on conceptual understanding, whereas FOOQ includes a higher proportion of discrete factual items. Beyond mean score differences, the observed shift from poor to good knowledge categories further underscores the relevance of the intervention effect at a clinically interpretable level. However, the interpretation of knowledge retention is limited by the absence of follow-up data in the control group, making it difficult to distinguish between sustained intervention effects and potential testing effects.

From a clinical perspective, video-based education offers several advantages. Standardized, guideline-based content ensures that each patient receives the same information, minimizing the risk of bias. It is a scalable measure and can be received by a wide number of patients simultaneously. Patients need minimal resources to consume the content. Clinical use can be pragmatic – for example, patients can watch educational content as preparatory material before physician appointments to create a knowledge base that facilitates and may improve (but not replace) face-to-face educational discussions.

Future research is needed to evaluate whether repeated or reinforced exposure to intervention content enhances the learning experience and which modalities can lessen the barrier between knowledge and clinical implementation. Measurable clinical outcome parameters such as treatment adherence and initiation or screening uptake could be used to determine the effect on clinically relevant outcomes, which were not assessed in this study. Additionally, studies incorporating longer follow-up periods and more diverse populations would further clarify the role of digital education in osteoporosis care pathways.

### Limitations

A formal sample size calculation was not performed prior to the study, which limits the ability to assess whether the study was adequately powered to detect differences, particularly in subgroup or follow-up analyses.

Follow-up data were available for only a subset of participants in the intervention group, with a relatively high loss to follow-up. This may have introduced selection bias, as participants who completed follow-up assessments could differ systematically from those lost to follow-up. In addition, no follow-up assessment was performed in the control group, which limits the interpretation of knowledge retention and prevents differentiation between sustained intervention effects and potential testing effects. The relatively short follow-up period further limits conclusions regarding long-term knowledge retention. The present findings therefore reflect only immediate and short-term effects of the intervention and do not allow inference on sustained knowledge retention over longer periods.

Due to the anonymous study design, individual-level linkage between baseline and follow-up responses was not possible. Consequently, follow-up analyses were restricted to unpaired group-level comparisons. This limits the ability to assess individual knowledge changes over time.

Participants were recruited through an online panel, which may limit the generalizability of the findings. Individuals who voluntarily participate in online health-related surveys may be more health-conscious or digitally literate than the general population. As a result, baseline knowledge levels and responsiveness to a video-based intervention may differ in other settings, such as older or less digitally engaged populations.

In addition, knowledge was assessed using self-administered questionnaires, which may be subject to response bias and test–retest effects, particularly in the short-term follow-up assessment. Although validated instruments were used, repeated exposure to similar items may have influenced performance independently of true knowledge acquisition.

Finally, the study focused exclusively on knowledge-related outcomes. While increased knowledge represents a prerequisite for informed decision-making, the present study did not assess downstream clinical outcomes such as behavioral change, treatment initiation, adherence, or fracture risk reduction.

## Data Availability

The datasets generated and analyzed during the current study are not publicly available due to privacy and data protection regulations but are available from the corresponding author on reasonable request.
